# Storm drains as larval development and adult resting sites for *Aedes aegypti* and *Aedes albopictus* in Salvador, Brazil

**DOI:** 10.1186/s13071-016-1705-0

**Published:** 2016-07-27

**Authors:** Igor Adolfo Dexheimer Paploski, Moreno S. Rodrigues, Vánio André Mugabe, Mariana Kikuti, Aline S. Tavares, Mitermayer Galvão Reis, Uriel Kitron, Guilherme Sousa Ribeiro

**Affiliations:** 1 Centro de Pesquisas Gonçalo Moniz, Fundação Oswaldo Cruz, Ministério da Saúde, Salvador, BA Brazil; 2 Instituto de Saúde Coletiva, Universidade Federal da Bahia, Salvador, BA Brazil; 3 Universidade Pedagógica de Quelimane, Quelimane, ZB Mozambique; 4 Faculdade de Medicina, Universidade Federal da Bahia, Salvador, BA Brazil; 5 Emory University, Atlanta, GE USA

**Keywords:** Epidemiology, Entomology, Arboviruses, Insect vectors, Disease vectors

## Abstract

**Background:**

Dengue (DENV), Chikungunya (CHIKV), Zika (ZIKV), as well as yellow fever (YFV) viruses are transmitted to humans by *Aedes* spp*.* females. In Salvador, the largest urban center in north-eastern Brazil, the four DENV types have been circulating, and more recently, CHIKV and ZIKV have also become common. We studied the role of storm drains as *Aedes* larval development and adult resting sites in four neighbourhoods of Salvador, representing different socioeconomic, infrastructure and topographic conditions.

**Results:**

A sample of 122 storm drains in the four study sites were surveyed twice during a 4-month period in 2015; in 49.0 % of the visits, the storm drains contained water. Adults and immatures of *Aedes aegypti* were captured in two of the four sites, and adults and immatures of *Aedes albopictus* were captured in one of these two sites. A total of 468 specimens were collected: 148 *Ae. aegypti* (38 adults and 110 immatures), 79 *Ae. albopictus* (48 adults and 31 immatures), and 241 non-*Aede*s (mainly *Culex* spp*.)* mosquitoes (42 adults and 199 immatures). The presence of adults or immatures of *Ae. aegypti* in storm drains was independently associated with the presence of non-*Aede*s mosquitoes and with rainfall of ≤ 50 mm during the preceding week.

**Conclusions:**

We found that in Salvador, one of the epicentres of the 2015 ZIKV outbreak, storm drains often accumulate water and serve as larval development sites and adult resting areas for both *Ae. aegypti* and *Ae. albopictus*. Vector control campaigns usually overlook storm drains, as most of the effort to prevent *Ae. agypti* reproduction is directed towards containers in the domicile environment. While further studies are needed to determine the added contribution of storm drains for the maintenance of *Aedes* spp. populations, we advocate that vector control programs incorporate actions directed at storm drains, including regular inspections and use of larvicides, and that human and capital resources are mobilized to modify storm drains, so that they do not serves as larval development sites for *Aedes* (and other) mosquitoes.

## Background

Arboviruses are a major and growing public health threat. Globally, dengue virus (DENV) is the most common arboviral infection, responsible for ~100 million symptomatic dengue cases annually [1]. More recently, chikungunya (CHIKV) and Zika viruses (ZIKV) have caused explosive outbreaks that spread from the Oceania and Asia to South and Central America [2].

Brazil reports the larger number of suspected DENV cases in the world (1.6 million cases in 2015), and simultaneous transmission of DENV, CHIKV and ZIKV was first documented in 2015 [3]. Following the 2015 ZIKV epidemic in Brazil, the virus rapidly spread to several countries in the continent, placing the rest of the world at risk for potential complications associated with infection, such as Guillain Barré syndrome in adults and congenital Zika syndrome in newborns [4–7].

DENV, CHIKV and ZIKV (as well as YFV) are transmitted to humans by *Aedes* spp*.* mosquitoes [8], mainly *Aedes aegypti*, which is common throughout the tropics and is particularly well-adapted to the urban environment [9]. *Aedes albopictus* is also a documented or potential vector of these arboviruses, and its geographic distribution extends to subtropical and temperate zones. Generally, *Ae. albopictus* is found in and around green areas within cities [9]. Vaccines against these arboviruses (with the exception of YFV) are not available for wide use. Therefore, vector control remains the key strategy to reduce arboviral transmission and subsequent human disease [1, 10–12].

The Brazilian National Dengue Control Program (NDCP) aims to improve case detection and vector control by, among others, strengthening epidemiological and entomological surveillance, as well as increasing accountability of householders to maintain an environment free of potential breeding sites [13]. Entomological surveillance is based on the inspection of homes and other structures and their surroundings for potential *Ae. aegypti* breeding sites, followed by their elimination or treatment with larvicides [14]. However, failure to consider and treat so-called cryptic breeding sites limits the effectiveness of control efforts [15–18]. Additionally, the NDCP has focused primarily on private households, often ignoring breeding sites located in public areas. Here we report findings from Salvador, one of the epicentres for the ZIKV epidemics in Brazil, highlighting the potential role of storm drains in the maintenance of *Ae. aegypti* and *Ae. albopictus* populations.

## Methods

### Study design

Based on circumstantial observations that storm drains could serve as a potential breeding site for *Aedes* mosquitoes in Salvador, we selected four study sites in different neighbourhoods of the city, and performed systematic surveys of the storm drains located in these sites, as described below. In all four sites, a path of 1–3 km along neighbourhood streets was arbitrarily chosen, and all storm drains on both sides of the streets were inspected. Each storm drain was inspected twice, between March and July 2015, approximately 30 days apart. All inspections were performed between 8:00 and 13:00 h. As the amount of accumulated rainfall in the days preceding the surveys could potentially influence the likelihood of finding mosquito immatures and adults in the storm drains, we tried to perform at least one of the two surveys in each storm drain on a day not preceded by a day with rainfall.

### Study sites

**Fig. 1:**
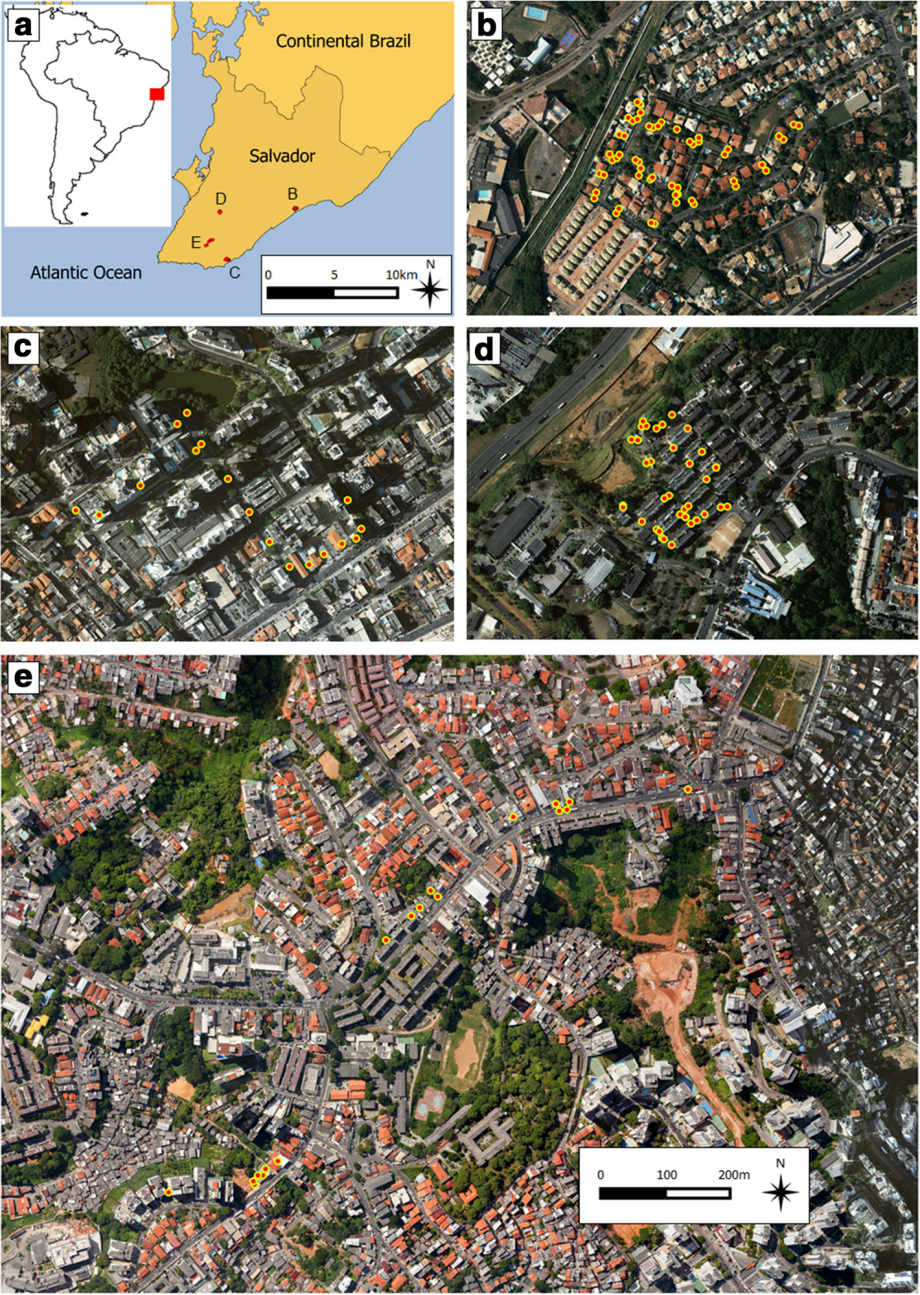
Location of the storm drains surveyed in Salvador, Brazil. **a** The red square in the South America map shows the location of Salvador in Brazil, and the red areas in the Salvador map show the locations of the four study sites within the city. **b**-**e** Aerial photograph of the four study sites, showing the location of the surveyed storm drains (red and yellow dots). **b**, **c**, **d** and **e** are to the same scale

Salvador is a city of 2.9 million inhabitants, located in north-eastern Brazil (Fig. 1a). The climate is tropical with an average annual rainfall of 2,150 mm and a mean temperature of 25 °C [19]. Since 2010, all four DENV serotypes co-circulate in Salvador [20], and in 2015, autochthonous transmission of CHIKV and ZIKV was confirmed in the city [3]. The four study sites (Piatã, Pituba, Cabula and Brotas) presented diverse socioeconomic, infrastructure and topographic conditions, and the distance among them ranged from 2.7 to 10 km (Fig. 1b-e, respectively).

Piatã (Fig. 1b) is a residential neighbourhood of high socioeconomic level with an estimated population of ~30,000 inhabitants. It is composed of several gated communities comprising individual homes with yards, whose appearance is similar to that of many US suburbs. Piatã is adjacent to the Atlantic coast, and the Piatã study site is ~200 m far from Piatã beach.

Pituba (Fig. 1c) is a neighbourhood of medium-high socioeconomic level (~50,000 inhabitants), characterized by tall residential buildings (frequently of > 10 floors) and a diverse network of street stores and other services. It is also situated by the coast, with the Pituba study site located ~150 m from Pituba beach.

Cabula (Fig. 1d) is a neighbourhood of medium-low socioeconomic level (~80,000 inhabitants), where small residential buildings (usually 3 floors high), often grouped into closed condominiums or housing complexes, coexist with a diverse range of services, such as the main general public hospital of Salvador, a public university and a large shopping center. Geographically, Cabula neighbourhood is centrally situated in Salvador, and the Cabula study site is located 5 km from the sea.

Brotas (Fig. 1e), (~80,000 inhabitants) is a neighbourhood of medium-low socioeconomic level, characterized by the presence of several commercial buildings, alongside with residential ones. The Brotas study site is 2.5 km far from the sea.

### Storm drains surveys

All storm drains locations were geocoded using a Garmin eTrex 10 portable global positioning system (GPS). Rainfall data were downloaded from the Brazilian National Institute of Metereology website [21], and the accumulated rainfall during the 7 days prior to the sampling of the drains was categorized as either ≤ 50 mm or > 50 mm, a rounded value of the median (54.4 mm) precipitation during the 7 days prior to the surveys.

In all four study sites, the surveyed storm drains were of the same general shape: a parallelepiped container ~100 cm long, ~30 cm wide and ~50 cm deep, covered with a metal or concrete grate, with discharge pipes near (but above) the bottom. The residual water volume in each storm drain was estimated by multiplying the height, width and length of the storm drain portion with accumulated water, and was categorized according to the median volume of accumulated water as ≤ 40 l or > 40 l. In addition, the storm drains were characterized according to potential for sunlight exposure (no shade, partial shade, or full shade); presence of organic material, such as leaves, fruits, wood or flowers in the accumulated water (yes or no); water odour (decomposed organic matter smell present or absent); and water turbidity (clear or turbid). Two inspectors performed all the storm drain surveys and used the same criteria to characterize the storm drains.

Prokopack aspirators [22] were used to collect adult mosquitoes in the storm drains. In storm drains containing water, a sample of one liter was collected from the surface of the storm drain water, in order to search for and collect immatures colonizing the drain. The water collection was performed using an entomological cup. The same two inspectors conducted all the mosquitos’ aspirations and water collections. Water samples containing immatures were transferred in Whirl-Pak® bags (Nasco) to the laboratory, where they were placed in mosquito cages (temperature ranging from 25 °C to 30 °C), maintained for 10 days to allow for development to adults, and inspected daily. All adult mosquitoes (either aspirated or reared from larvae/pupae were identified under a dissecting scope and classified according to morphological-based taxonomic keys into three groups: *Ae. aegypti, Ae. albopictus* or other (mostly *Culex* spp.).

### Statistical analysis

Data were recorded and managed using the REDCap electronic data capture tool [23], and statistical analysis were conducted using STATA 14 [24]. We described the storm drains characteristics by study site and survey period, including the proportions of drains with accumulated water, and among those containing water, the proportion with adult and immature *Aedes* mosquitoes. In addition, we estimated by study site and survey period the accumulated rainfall during the 7 days preceding each survey (or the mean rainfall precipitation in the 7 days preceding the survey if more than one day was needed to complete the site survey), and the mean volume of water in storm drains with water.

We classified the surveyed storm drains as either infested with *Ae. aegypti* (when adults and/or immatures were present), or as uninfested (when neither was present). Comparison of frequency of infestation between groups of storm drains was assessed using Chi-square test (*P* < 0.05). To associate characteristics of water-filled storm drains with the presence of *Ae. aegypti* infestation*,* we applied a bivariate logistic regression model, and variables with *P* ≤ 0.20 were included in a multivariable model; backward selection was used to obtain the final model which included all remaining variables associated with *Ae. aegypti* presence at *P* < 0.05.

## Results

A total of 122 storm drains were identified in the four study sites. All of them were surveyed twice, except for one in Cabula, one in Brotas and one in Pituba (where access was not possible due to cars parked on the drains), for a total of 241 inspections. The route length and number of drains identified in each of the sites were 1,687 m and 52 drains in Piatã; 1,787 m and 35 drains in Cabula; 3,123 m and 18 drains in Brotas; and 1,017 m and 17 drains in Pituba (Fig. 1).

We found accumulated water in nearly half (118; 49.0 %) of the 241 storm drain inspections. Storm drains with accumulated water were observed in the four study sites, but were much more common in Piatã and Brotas (Table 1). Of the 57 storm drains containing water during the first survey, 51 (89.5 %) contained water also in the second survey, while 10 (15.6 %) of the 64 drains that did not have water in the first survey had water in the second survey. The average estimated volume of water in the storm drains with water was 42.4 (standard deviation 28.7; range 0.52 to 214.5) liters.

**Table 1:** Findings from entomological surveys performed in storm drains of four neighbourhoods in Salvador, Brazil in 2015

Survey characteristics	Piatã		Cabula		Brotas		Pituba	
Survey date (day/month of 2015)	10 & 13/Mar	28 & 29/Mar	06 &12/May	27/Jul	11/Jun	29/Jul	14/Jul	31/Jul
Average precipitation (mm) during 7 days prior to survey	2.7	60.3	51.4	54.4	87.4	47.4	79.6	36.8
No. of surveyed storm drains	52	52	35	34	18	17	16	17
No. (%) of storm drains with accumulated water	38 (73.1)	48 (92.3)	5 (14.3)	2 (5.9)	11 (61.1)	9 (52.9)	3 (18.8)	2 (11.8)
Average volume (liters) of water	32.2	51.0	28.7	45.0	36.5	60.4	19.7	0.0
No. (%) of water-containing storm drains with larvae/pupae of								
*Aedes aegypti*	11 (28.9)	1 (2.1)	1 (20.0)	–	–	–	–	–
*Aedes albopictus*	–	–	2 (40.0)	1 (50.0)	–	–	–	–
Other mosquitoes	19 (50.0)	2 (4.2)	1 (20.0)	1 (50.0)	–	–	1 (6.3)	–
No. (%) of storm drains with adults of:				–	–	–	–	–
*Aedes aegypti*	8 (15.4)	2 (3.8)	1 (2.9)	–	–	–	–	–
*Aedes albopictus*	–	–	8 (22.9)	1 (2.9)	–	–	–	–
Other mosquitoes	12 (23.1)	8 (15.4)	9 (25.7)	1 (2.9)	–	–	–	–

Adult and immature *Ae. aegypti* were captured in two of the four sites, Piatã and Cabula. Adult and immature *Ae. albopictus* were captured only in Cabula (Table 1). We captured 468 specimens, of which 148 were *Ae. aegypti* (38 adults and 110 immatures), 79 were *Ae. albopictus* (48 adults and 31 immatures), and 241 were non-*Aede*s (mainly *Culex* spp.) mosquitoes (42 adults and 199 immatures) (Table 2). With the exception of six *Aedes albopictus* adults, all the remaining *Aedes* specimens were captured in storm drains containing water. In the storm drains where adult *Ae. aegypti* were captured, the median number caught was 1 (range 1–12). In the storm drains where immature *Ae. aegypti* were captured, the median number was 3 (range 1–49). In contrast, in the storm drains where adult *Ae. albopictus* were captured, the median number was 2 (range 1–35), and in the storm drains where immature *Ae. albopictus* were captured, the median number was 9 (range 3–19).

**Table 2:** Total number of mosquitoes (adults and immatures) captured during storm drain surveys in four neighbourhoods of Salvador, Brazil in 2015

Neighbourhood	Specimens					
	*Ae. aegypti*		*Ae. albopictus*		Other mosquitoes	
	Adults	Larvae and pupae	Adults	Larvae and pupae	Adults	Larvae and pupae
	M/F		M/F		M/F	
Piatã	17/20	109	0	0	17/24	167
Cabula	0/1	1	14/34	31	1/0	24
Brotas	0	0	0	0	0	0
Pituba	0	0	0	0	0	8
Total	38	110	48	31	42	199

*Abbreviations*: *M* male, *F* female

Immature mosquito predators, such as tadpoles or any other natural predators, were not found in any of the storm drains. *Aedes aegypti* infestation in storm drains containing non-*Aedes* mosquitoes was more frequent (41.8 % of inspections) than in storm drains without non-*Aedes* mosquitoes (5.8 %) (*χ*^*2*^ *= 23.2, df* = 1, *P* < 0.001) (Table 3). In addition, *Ae. aegypti* infestation was more commonly observed when the water volume was ≤ 40 l (24.6 %), than when it was > 40 l (6.9 %) (*χ*^*2*^ *= 6.8, df* = 1, *P* = 0.009), and when the accumulated rainfall precipitation in the preceding 7 days was ≤ 50 mm (28.3 %) compared to when it was > 50 mm (4.6 %) (*χ*^*2*^ *= 12.7, df* = 1, *P* < 0.001). Other characteristics, such as presence of shade, organic matter, water turbidity and water odour were not associated with presence of *Ae. aegypti* infestation*.*

**Table 3:** Factors associated with *Aedes aegypti* (adults or immatures) presence in 118 storm drains with accumulated water, Salvador, Brazil, 2015

Characteristic	No. with available data	No. with *Aedes aegypti* (%)	Crude OR (95 % CI)	Adjusted OR (95 % CI)
Organic matter				
No organic matter	22	3 (13.6)	1	
With organic matter	96	15 (15.6)	1.2 (0.3–4.5)	
Shade				
No shade	64	9 (14.0)	1	
Indirect shade	37	5 (13.5)	1.0 (0.3–3.1)	
Direct shade	17	4 (23.5)	1.9 (0.5–7.1)	
Turbidity of water^a^				
No turbid	61	7 (11.5)	1	
Turbid	47	11 (23.4)	2.4 (0.8–6.7)	
Water odor^a^				
No odor	51	10 (19.6)	1	
With odor	59	8 (13.6)	0.6 (0.2–1.8)	
Non-*Aedes* mosquitoes^b^				
Absent	87	5 (5.8)	1	1
Present	31	13 (41.8)	11.8 (3.8–37.4)	7.8 (2.3–25.8)
Water volume^a^				
≤ 40 l	57	14 (24.6)	1	
> 40 l	58	4 (6.9)	0.2 (0.1–0.7)	
Precipitation during the previous 7 days				
≤ 50 mm	53	15 (28.3)	1	1
> 50 mm	65	3 (4.6)	0.1 (0.0–0.5)	0.2 (0.1–0.8)

^a^Data on water turbidity, odor and volume were not collected for 10, 10 and 3 storm drains, respectively

^b^Non-*Aedes* mosquitoes: includes adult mosquitoes and immatures

In the multivariable model, presence of non-*Aedes* mosquitoes remained significantly associated with increased odds of *Ae. aegypti* infestation (OR: 7.8; 95 % CI: 2.3–25.8) (Table 3). Additionally, > 50 mm of accumulated rainfall during the 7 days prior to the survey was associated with significantly reduced odds of finding *Ae. aegypti* (OR: 0.2; 95 % CI: 0.05–0.8) (Table 3).

## Discussion

We demonstrated that in Salvador, one of the largest urban centers in Brazil, and an epicentre of the recent ZIKV outbreak, storm drains often accumulate water and serve as larval development sites and adult resting areas for both *Ae. aegypti* and *Ae. albopictus*. In addition, we found that the presence of non-*Aedes* mosquitoes in storm drains (for which storm drains are a well-documented habitat, especially for *Culex pipiens/quinquefasciatus* species [25–28] was associated with presence of *Ae. aegypti*.

Previous studies have highlighted the potential contribution of specific sites to the overall adult mosquito population. In Australia, out of 1,349 premises inspected for *Ae. aegypti* presence, two were responsible for 28 % of all immature forms, thus reinforcing the concept of “key containers” (i.e. specific sites that are responsible for a large proportion of mosquitoes found in as area) [29]. In our study, because it was not possible to collect all the water from the surveyed storm drains, the number of immature forms that we report underestimates the total number of larvae and pupae present in this aquatic habitat. However, the finding of as many as 49 *Ae. aegypti* and 19 *Ae. albopictus* larvae/pupae in one liter of water, while the median volume of residual water on inspected storm drains was 42.4 liters illustrates the potential of storm drains to serve as key development sites for *Aedes* mosquitoes.

Historically, dengue prevention campaigns in Brazil have focused on households, aiming to identify and eliminate *Aedes* breeding sites, or, when source reduction is not possible, to treat the water with larvicides. In addition, large public campaigns are used to mobilize the public to combat the vector. These campaigns emphasize the elimination of *Ae. aegypti* breeding sites in citizens’ households. Consequently, breeding sites located in public areas, and especially non-container breeding sites, are often ignored.

Non-household breeding sites, such as storm drains [30], manholes, sub-surface catch basins [15] and non-disposable containers [31, 32] have been previously identified as important habitats for *Ae. aegypti* and other mosquitoes in several studies. In Mexico, in an intensive mosquito capture effort aiming to understand the relative importance of different containers as larval habitats, all 15 storm drains identified in the study area contained residual water, and 60 % of them were populated by *Ae. aegypti* larvae and adults, in contrast to seven containers found during regular house inspections in the area, of which only one contained *Ae. aegypti* larvae [15]. The authors also estimated the number of adults produced per day in the storm drains to be 12, in contrast to zero from the regular containers found during house inspections [15]. In Guadalajara de Buga, Colombia, a quasi-experimental study showed a reduction in the frequency of larval infestation of storm drains and a sharp decline (81 %) in human dengue cases following chemical control of larvae in all storm drains of the city; no such decline was detected in a control community, where no intervention was performed [33]. Another study, from Australia, demonstrated that the mean distance between dengue seropositive people and the nearest subterranean container (mostly wells and manholes) was shorter than for randomly selected controls. Additionally, the prevalence of antibodies for dengue in residents living < 160 meters away from a well or service manhole was 2.5 times higher than in residents living further away [17]. Given that storm drains are ubiquitous in the urban setting, and the accumulated evidence pointing to their potential contribution to arbovirus spread through the urban environment, storm drains monitoring needs to be prioritized.

Targeting “cryptic” breeding sites (including storm drains) for surveillance and control needs to become an essential part of vector control programs in Salvador and other urban areas. This is particularly relevant for Brazil, not only in light of the recent outbreaks of ZIKV and CHIKV, but also because the national vector control programs traditionally rely on a household level index, the LIRA (larval index rapid assay), which does not incorporate surveys of public spaces.

Our study is subject to several limitations. Although it is likely that our findings are valid for much of Salvador and other tropical urban sites, it was restricted to two surveys in four areas of just one city. We also did not investigate other potential alternative larval development sites within the four study sites and, therefore, could not estimate the relative contribution of storm drains to *Aede*s populations in each of the sites. In addition, our surveys may have underestimated the numbers of adults and immatures, because the sampling periods fell within the rainy season. Finally, our measurements were made during the morning and do not necessarily reflect the rest of day; this is especially relevant for the daytime active *Aedes* mosquitoes, whose adults may use storm drains even more during the night. Overall, our study needs to be extended both spatially and temporally in order to assess the wider role of storm drains (and other ignored larval sites) in *Aedes* mosquitoes development, and their contribution to arboviruses transmission.

Despite these limitations, our findings can already be applied to guide vector control interventions. We have shared our results with the Zoonosis Control Center at the Municipal Secretary of Health, the administrative unit responsible for the vector control program in Salvador, and with the community leaders and residents of the two closed condominiums where we conducted the study. In one of them, the local association of homeowners agreed on the priority of drying the storm drains, and are paying themselves for filling the bottom of the storm drains with concrete, in order to prevent the accumulation of water in them.

## Conclusions

We have shown that storm drains can serve both as important larval development and as adult resting sites for *Ae. aegypti* and *Ae. albopictus*, which can complete a large portion of their life-cycle in this hospitable and protected environment. We recommend that efforts to control *Aedes* mosquitoes and outbreaks of DENV, CHIKV, and ZIKV take into account storm drains as potential sites for vectors reproduction. Traditional and novel strategies to control mosquito population in these aquatic sites, including (but not limited to) the use of insect growth regulators (e.g. methoprene) [34], *Bacillus thuringiensis israelensis* (BTI) [35] and residual insecticides, needs to be evaluated. However, as an ultimate solution, we advocate for a better design of storm drains [36] that restricts the long-term accumulation of water.

## Abbreviations

BTI, *Bacillus thuringiensis israelensis;* CHIKV, Chikungunya virus; DENV, Dengue virus; LIRA, larval index rapid assay; NDCP, Brazilian National Dengue Control Program; YFV, yellow fever virus; ZIKV, Zika virus
